# Coupling Hydroxyapatite Nanocrystals with Lactoferrin as a Promising Strategy to Fine Regulate Bone Homeostasis

**DOI:** 10.1371/journal.pone.0132633

**Published:** 2015-07-06

**Authors:** Monica Montesi, Silvia Panseri, Michele Iafisco, Alessio Adamiano, Anna Tampieri

**Affiliations:** Institute of Science and Technology for Ceramics, National Research Council, Faenza, Ravenna, Italy; Faculté de médecine de Nantes, FRANCE

## Abstract

Lactoferrin (LF) is an interesting glycoprotein in the field of bone biology for its regulatory effect on cells involved in bone remodeling, that results compromised in several pathological conditions, as osteoporosis. In a previous study we observed that the coupling of LF and biomimetic hydroxyapatite nanocrystals (HA), a material well-known for its bioactivity and osteoconductive properties, leads to a combined effect in the induction of osteogenic differentiation of mesenchymal stem cells. On the basis of this evidence, the present study is an extension of our previous work aiming to investigate the synergistic effect of the coupling of HA and LF on bone homeostasis. Biomimetic HA nanocrystals were synthesized and functionalized with LF (HA-LF) and then pre-osteoblasts (MC3T3-E1) and monocyte/macrophage cells lines (RAW 264.7), using as osteoclastogenesis *in vitro* model, were cultured separately or in co-culture in presence of HA-LF. The results clearly revealed that HA and LF act in synergism in the regulation of the bone homeostasis, working as anabolic factor for osteoblasts differentiation and bone matrix deposition, and as inhibitor of the osteoclast formation and activity.

## Introduction

Bone homeostasis is maintained *via* an equilibrium of bone resorption and bone formation and the intercellular crosstalk, that occurs among bone cells, is a critical process for the maintenance of normal bone structure [1]. Osteoporosis is the most worldwide, chronic, progressive and multifactorial bone disease with a very high prevalence in humans older than 50 years, caused by an imbalance between the activities of osteoclasts (OCLs) and osteoblasts (OBs) [2]. The main clinical consequences of osteoporosis are bone fractures, which often lead to patient disability or even death, thus this disease is consider the major cause of morbidity and health expenditure in aging populations [3, 4].

Nowadays, no satisfactory solution exists to the problem of bone weakening caused by osteoporosis, therefore, investigation and exploitation of innovative therapeutic strategies aimed to reduce the clinical complications of this disease are of great scientific and socio-economic interest.

In the last decades, the innovation in nanotechnology and the development of natural-derived biomaterials have influenced therapeutic approaches in different areas of medicine and in particular the production of advanced biomaterials for orthopedic applications [5, 6]. Several efforts have been invested to achieve new nanostructured bioactive materials for bone substitution exhibiting structural properties comparable with those of natural healthy bone.

Nanometer-sized hydroxyapatite (HA) based materials, mimicking the dimension and composition of the mineral phase of natural bone, have drawn great attention in regenerative medicine and tissue engineering for their excellent properties of biocompatibility, osteointegration and ability to act as carriers of drugs, proteins, genes and other bioactive molecules that can be absorbed or selectively linked to their surface [7–11].

Lactoferrin (LF) is an 80-kDa iron-binding glycoprotein which has been established as a potent anabolic molecule for the skeleton [12] due to its ability to increase OBs proliferation, survival, and differentiation and moreover to decrease OCLs formation [13–15]. Although the molecular mechanisms underlying LF action are still largely unknown, the interest for this bioactive molecule is recently grown and its application in bone disease treatments, alone or in combinations with other systems, is greatly emerging [16–18].

In this scenario, the functionalization of biomimetic HA nanocrystals with LF could be an innovative strategy to set up a novel functional biomaterial aiming to reduce the imbalance in bone homeostasis occurring in osteoporosis. The rationale behind the development of HA-LF system is that the association of the peculiar properties of each components, such as the well-known osteoconductive properties of biomimetic HA and its role in the induction of the osteogenetic related genes expression, with the bioactivity of LF [14, 19], could act in synergism to promote the activation of bone-forming OBs and to inhibits bone—resorbing OCLs. In previous studies we have demonstrated that the conjugation of HA with LF is strong and stable and leads to a combined effect in the induction of osteogenic differentiation of mesenchymal stem cells (MSCs) [20, 21]. Therefore, this paper can be viewed as an extension of our previous work. Here the behavior of OBs and OCLs cells grown in direct contact with HA-LF was tested *in vitro*. Moreover, the potential role of HA-LF in the regulation of bone intercellular cross-talk, focusing on the pathway involved in the inhibition of the OCLs activity mediated by OBs, was investigated in a co-culture model.

## Materials and Methods

### Preparation of HA nanocrystals and functionalization with LF

HA nanocrystals were prepared and characterized as previously described [19]. HA nanocrystals were functionalized with LF according to the procedure reported by Montesi et al [20]. Briefly, 50 mg of HA were mixed with 7.5 ml of LF solution (0.5 mg/ml) in HEPES buffer at pH 7.4 (0.01 M HEPES, 0.15 M NaCl) at 37°C for 24 h, in order to obtain a 6.6 wt% of LF loaded onto HA nanocrystal surface. The solid was washed twice with 1 ml of ultrapure water, recovered by centrifugation and then freeze dried with a freezing ramp from 25°C to –40°C and a heating ramp from –40°C to 25°C under vacuum conditions (P = 0.20 mbar). The amount of attached LF onto HA was calculated from the difference between the LF concentration in solution before and after the adsorption on HA by UV-Vis spectroscopy (λ = 280 nm, ε = 92 000 M^-1^ cm^-1^) using a Cary Bio spectrophotometer (Varian, Palo Alto, CA, USA) and by thermogravimetric analysis (TGA) on freeze dried samples after washings using a Thermal Analysis SDT Q 600 (TA Instruments, New Castle, DE), as previously reported [20]

### Cell cultures

Pre-osteoblast cell line MC3T3-E1 Subclone 14, obtained from ATCC cell bank (Manassas, VA, USA), was used as model of OBs [22]. MC3T3-E1 were cultured in αMEM containing ribonucleosides, deoxyribonucleosides (GIBCO), 50 μg/ml of ascorbic acid, L-glutamine, 10% FBS and 100U/ml penicillin/streptomycin.

Murine monocyte/macrophage cell line RAW 264.7 obtained from ATCC cell bank (Manassas, VA, USA), was used as model of osteoclastogenesis [23, 24]. RAW 264.7 cells were cultured in DMEM high glucose (GIBCO), 10% FBS and 100U/ml penicillin/streptomycin. To initiate OCLs differentiation, 25 ng/mL soluble Receptor Activator for Nuclear Factor k B Ligand (sRANKL, Sigma- Aldrich, St Louis, MO, USA) was added.

For the experiments, both the cell lines were plated at a density of 1.5×10^3^ cells/cm^2^ in 6 and 24-well plates, and 24 hours after seeding the samples were added to the culture. Both the cell lines were also cultured for 7 and 14 days in direct contact with HA-LF at 10 μg/ml concentration of attached LF. Moreover, the equivalent amounts of free LF and HA were tested, and a group of cells only was used as a control (Table 1).

**Table 1:** Samples identification.

Samples	LF loaded (wt%)	LF tested (μg/ml)	HA tested (μg/ml)
**HA-LF**	6.6	10	141.5
**HA**	-	-	141.5
**LF**	-	10	-
**Cells only**	-	-	-

Labelling of the samples, weight percentage (wt%) of LF loaded onto HA and LF and HA concentration (μg/ml) of the samples tested in the study.

Cellular behaviour in OBs and OCLs co-culture was evaluated. RAW 264.7 cells at a concentration of 1.5×10^3^ cells/cm^2^ were seeded in 0.4 μm pore size 24-well inserts (Millipore, Ireland) and co-cultured with MC3T3-E1 cells (1.5×10^3^ cells/cm^2^) seeded on 24-well plate. The day after the seeding the samples were added to the MC3T3-E1 cells seeded on 24-well plate and the co-culture was grown for 7 days in OBs:OCLs culture medium (50:50).

### Cell viability and apoptosis assay

Live/Dead assay kit (Invitrogen) was performed according to manufacturer's instructions. This assay is based on the simultaneous determination of live and dead cells with two probes, Calcein acetoxymethyl (Calcein AM) and Ethidium homodimer-1 (EthD-1), measuring recognized parameters of cell viability, intracellular esterase activity and plasma membrane integrity respectively [25].

Briefly, at 7 and 14 days MC3T3-E1 cells were washed with 0.01M PBS for 5 min and incubated with Calcein AM 2 μM plus EthD-1 4 μM for 15 min at 37°C in the dark, the samples were rinsed in 0.01M PBS. The live/dead cells ratio was determined by quantifying the number of cells in 3 random fields of view per sample at the same magnification. One sample per group was analysed.

MC3T3-E1 cell apoptosis was assessed at day 7 and 14 by counting cells, previously fixed with 4% (w/v) paraformaldehyde for 15 min and stained with DAPI (Invitrogen),exhibiting chromatin condensation and/or nuclear blebbing [26, 27]. Positive cells were quantified by visualizing 4 random fields of view per sample at 20X magnification and counting the number of positive cells *versus* the total number of cells per image. All the images were acquired by an Inverted Ti-E fluorescence microscope (Nikon).

### TRAP staining

After 7 days, the differentiated RAW 264,7 cells were fixed and stained for tartrate-resistant acid phosphatase (TRAP activity), a marker of osteoclast differentiation and resorbing activity [28], following the kit protocol, Acid Phosphatase, Leukocyte (Sigma- Aldrich, St Louis, MO, USA). Briefly, cells were fixed in a solution containing 37% formalin, acetone and citrate solution for 1 minute at room temperature, and rinsed thoroughly in deionized water. Than the cells were incubated in a solution containing Naphthol AS-Bl phosphoric acid, 12.5 mg/ml, Fast garnet GBC base, 7.0 mg/ml, in 0.4 mol/l hydrochloric acid with stabilizer, Acetate buffer, 2.5 mol/l, pH 5.2, Sodium nitrite, 0.1 mol/l and L(+)-Tartrate buffer, 0.335 mol/l, pH 4.9, for 1 hour at 37°C in controlled humidity and protected from light. After 1 hour, the cells were rinsed thoroughly in deionized water and counterstained for 2 minutes in Hematoxylin Solution. Images were acquired by an Inverted Ti-E fluorescence microscope (Nikon).

### Dosage of soluble factors RANKL and OPG (ELISA assay)

After 7 days of culture, the culture media derive from the RAW 264.7 and MC3T3-E1 co-culture were tested following the kit protocol instructions. Briefly, the culture media were centrifuged to remove particulates and one hundred microliters were tested. The enzyme-linked immunosorbent assay (ELISA) is a common method used to quantify the presence of soluble factors, and it is based on capacity of specific antibodies of recognise selectively a sequence of the factor detected. The presence of the factor is detected by using a secondary antibody peroxidase conjugated; the adding of the enzymatic substrate leads to the production of a visible signal, detected with spectrophotometry at λ_max_ of 450 nm. The total amounts of soluble factors production were converted to picograms per millilitre (pg/ml) using the standard curve and Osteoprotegerin/Receptor activator of nuclear factor kappa-B ligand (OPG/RANKL) ratio was calculated. RANKL was detected by using Mouse OPG ELISA Kit and Mouse TNFSF11/RANKL ELISA Kit (Boster Biological Technology, Fremont, CA). Co-culture medium with the addition of RANKL was evaluated and subtracted from RANKL samples values.

### Quantitative real-time polymerase chain reaction (qPCR)

At day 7 and 14, cells grown in presence of the samples and cells only, used as a calibrator, were homogenized and total RNA extraction was performed by use of the Tri Reagent, followed by the Direct-zol RNA MiniPrep kit (Zymo Research) according to manufacturer's instructions. RNA integrity was analysed by native agarose gel electrophoresis and quantification performed by the Qubit 2.0 Fluorometer together with the Qubit RNA BR assay kit, following manufacturer's instructions (Invitrogen). Total RNA (500 ng) was reverse transcribed to cDNA using the High-Capacity cDNA Reverse Transcription Kit, according to manufacturer's instructions (Applied Biosistem). Quantification of OBs gene expression for integrin-binding sialoprotein (IBSP, Mm00173726), Osterix (Mm04209856) and quantification of OCLs gene expression for Integrin β3 (Itgβ3, Mm00443980), Oscar (Mm00558665) and Cathepsin K (CtsK, Mm0048403) was performed by use of the StepOne Real-Time PCR System (Applied Biosystems); glyceraldehyde 3-phosphate dehydrogenase (GAPDH, Mm99999915) was used as housekeeping gene. Experiment was done in triplicate, using three technical replicates for each sample. Data were collected using the OneStep Software (v.2.2.2) and relative quantification was performed using the comparative threshold (Ct) method (ΔΔCt), where relative gene expression level equals 2^−ΔΔCt^ [29].

### Statistical analysis

Results were expressed as Mean± Standard Error (SEM) plotted on graphs. Analysis of the effect of HA-LF on cell culture (cell viability and apoptosis) was made by two-way ANOVA, followed by Bonferroni's post-hoc test. Analysis of the effect of the HA-LF on gene expression was made by one-way ANOVA, followed by Tukey's Multiple Comparison Test. Statistical analyses were performed by the GraphPad Prism software (version 5.0), with statistical significance set at p≤0.05.

## Results

### Positive effect of HA-LF system on OBs behaviour

In order to assess the effect of HA-LF on the OBs viability after 7 and 14 days of culture, the quantification of the cells exhibiting cytotoxic events, distinguishable by the damaged membranes and by chromatin condensation/nuclear blebbing, has been carried out.

**Fig 1:**
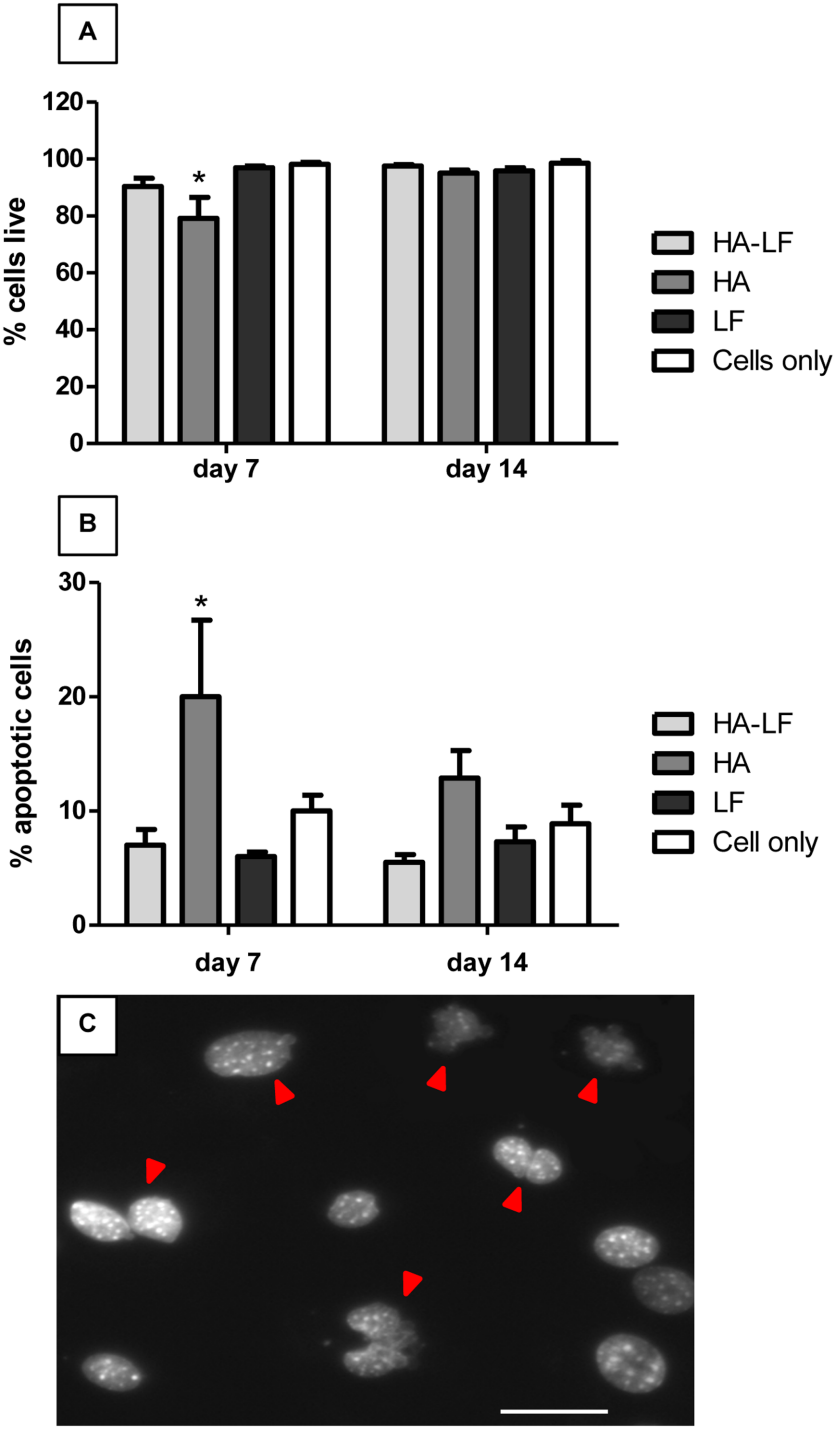
OBs viability and apoptosis. (A) shows the percentage of live OBs respect to the total cells counted and (B) shows the percentage of apoptotic OBs respect to the total cells counted. Mean and standard error (n = 3) represented as the percentage of the total counted cells, after 7 and 14 days of culture in direct contact with all the tested samples. Statistical significant differences among the samples are indicated in both graphs: *p≤0.05. (C) Different examples of nuclear fragmentation in OBs stained with DAPI are indicated with red arrows. Scale bar 50 μm.

The ratio of live cells showed no significant difference among all the samples tested except for HA at day 7 that showed a statistical significant decrease of live cells compared to all the other samples (p<0.05) (Fig 1A). After 14 days of culture this difference disappeared and the percentage of cells live revealed no differences among the samples and the cells only used as control (Fig 1a). Moreover, also the percentage of apoptotic OBs was significant higher in HA sample after 7 days of culture (p <0.05) compared to all the samples tested (Fig 1b and 1c). After 14 days of culture apoptotic cells in HA samples decreased, while no changing were found in the other samples (Fig 1b).

**Fig 2:**
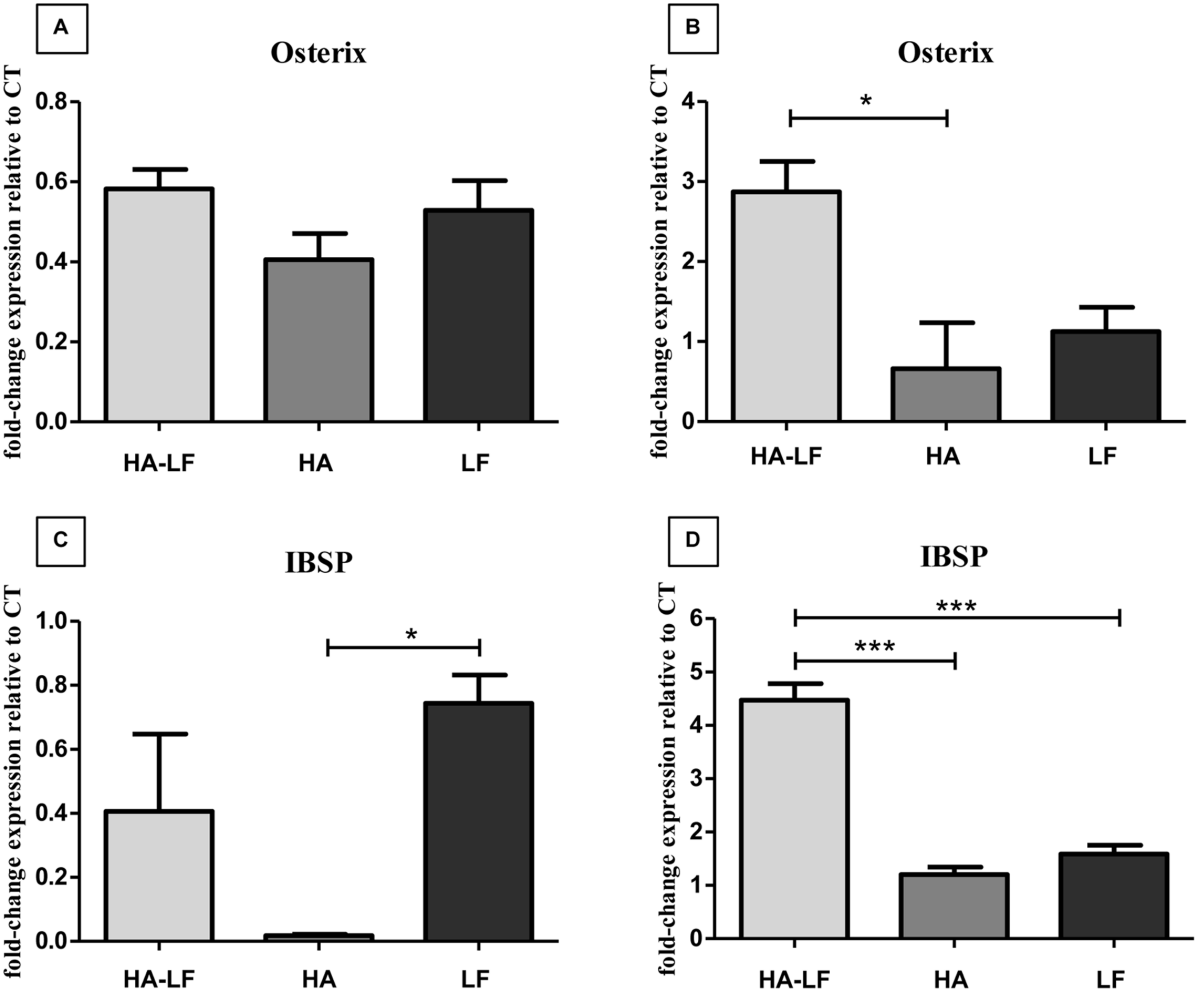
OBs gene expression analysis. (a) and (c) Relative quantification (2^-ΔΔCt^) of gene expression after 7 and (b) and (d) 14 days of OBs cultured in direct contact with all the tested samples. The graph shows the average and standard error of the technical triplicate of Osterix and IBSP, respect to the expression of the cells only, used as a control. Statistical significant differences among the samples are indicated in the graphs: *p≤0.05 and ***p≤0.001.

The quantification of Osterix and IBSP mRNA level showed that after 7 days cells expressed a basal, or lower level of both the genes compared to the cells only (Fig 2a and 2c). Instead, after 14 days, HA-LF significantly upregulated the expression of both these genes (Fig 2b and 2d); in detail, the sample HA-LF significantly increased the Osterix relative expression compared to HA sample (p<0.05) and the IBSP relative expression compared to both HA and LF samples (p<0.001).

### Effect of HA-LF on OCLs differentiation and activity

In order to evaluate the behaviour of OCLs grown in direct contact with the HA-LF, osteoclasts formation has been assessed by TRAP staining and relative quantification of Oscar, Itgβ3 and CtsK genes were evaluated by qPCR.

**Fig 3:**
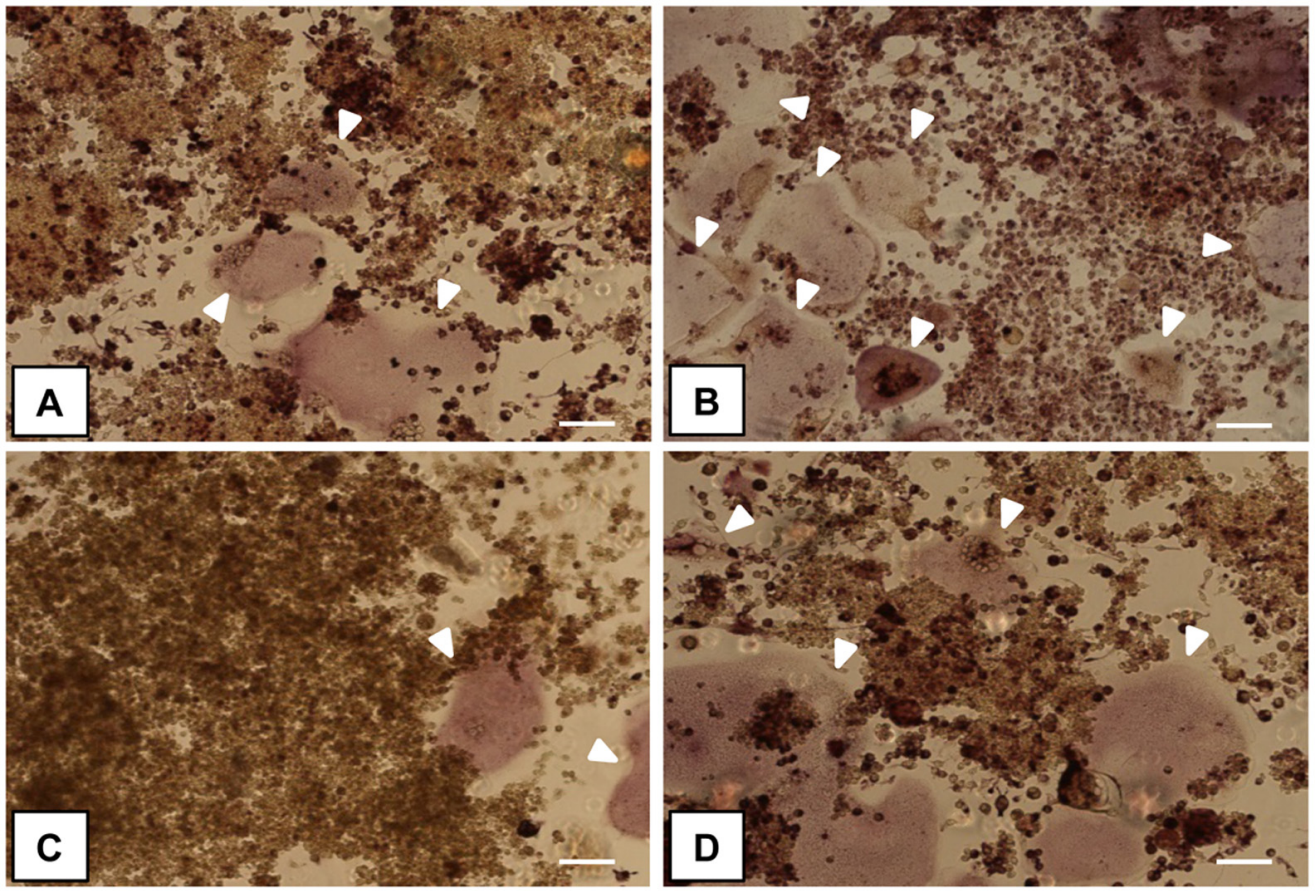
Multinucleated TRAP^+^ OCLs. The images show sRANKL-treated RAW264.7 cells cultured in direct contact with the samples: (A) HA-LF, (B) HA, (C) LF and (D) cells only, respectively. White arrows indicate large and multinucleated cells positive for tartrate-resistant acid phosphatase (mature OCLs). Scale bar 100 μm.

The qualitative evaluation of TRAP staining indicated that the presence of LF, both attached onto HA and free (Fig 3a and 3c), reduced the OCLs formation identifiable as large multinucleated cells positive for TRAP activity, compared to HA and cells only samples (Fig 3b and 3d).

**Fig 4:**
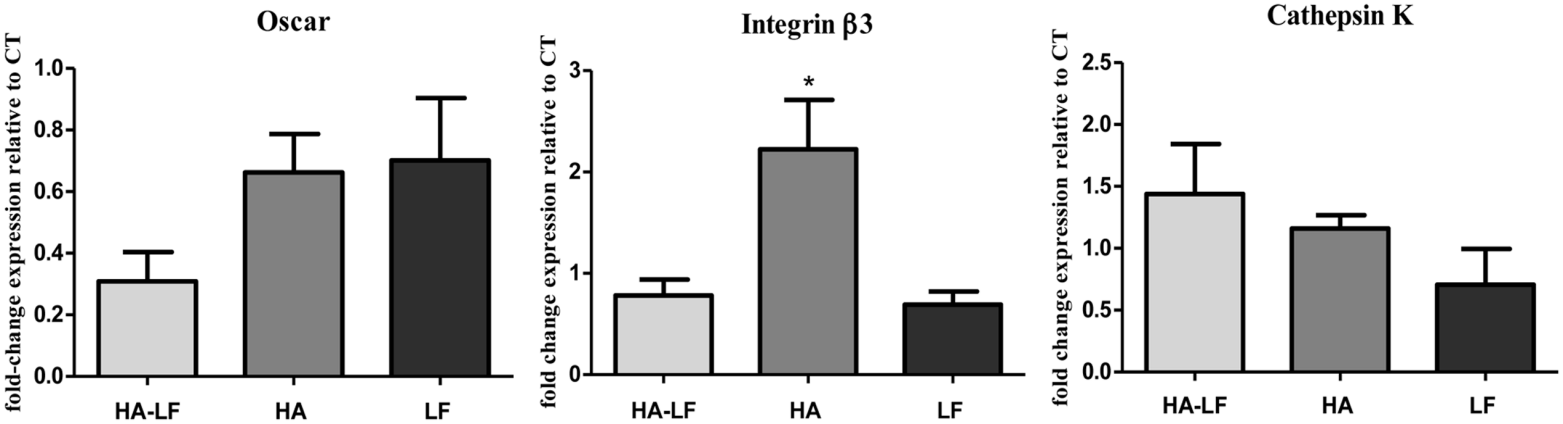
OCLs gene expression analysis. Relative quantification (2^-ΔΔCt^) of gene expression after 7 days of OCLs cultured in direct contact with all the tested samples. Average and standard error of the technical triplicate of Oscar, Itgβ3 and CtsK, respect to the expression of the cells only, were indicated. Statistical significant difference among the samples is indicated in the graph: *p≤0.05.

The quantification of OCLs related genes showed no significant differences in Oscar and CtsK relative expression among the tested samples (Fig 4). Conversely, the relative expression of Itgβ3 was significantly lower in HA-LF and LF compared to HA (p<0.05) (Fig 4).

### Effect of HA-LF on OCLs regulation mediated by OBs

The crosstalk between OBs and OCLs has been evaluated in a co-culture *in vitro* model, in order to clarify the role of HA-LF in OCLs inhibition mediated by OBs.

**Fig 5:**
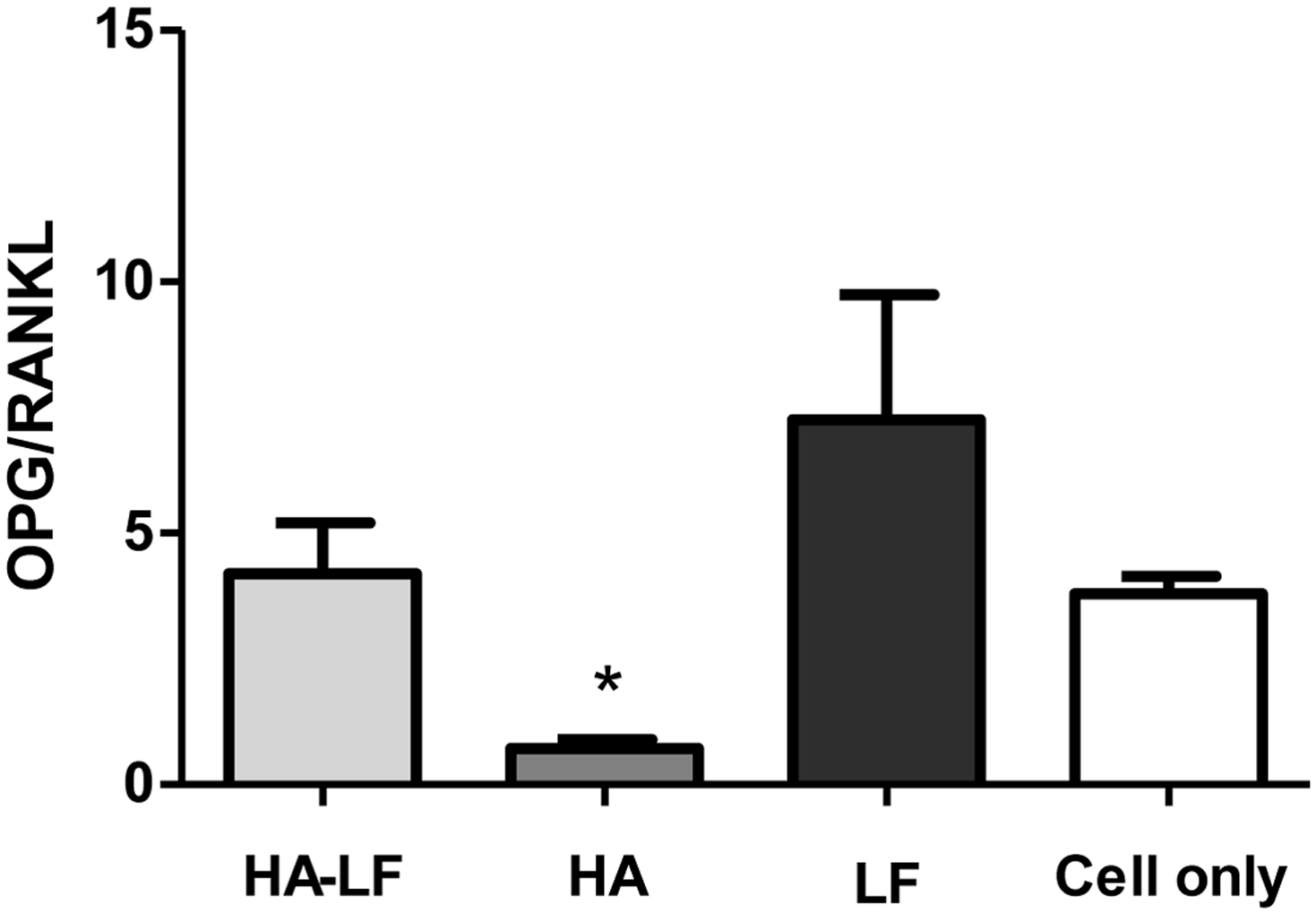
OPG/RANKL ratio in OBs/OCLs co-culture. In the graph is reported the ratio of the soluble factors measured by ELISA kit. Mean and standard error of three replicates are shown. Statistical significant differences among the samples are indicated in the graph: *p≤0.05.

The ratio of OPG/RANKL (Fig 5) was significantly lower in HA samples compared to HA-LF, LF and Cells only (p<0.05).

**Fig 6:**
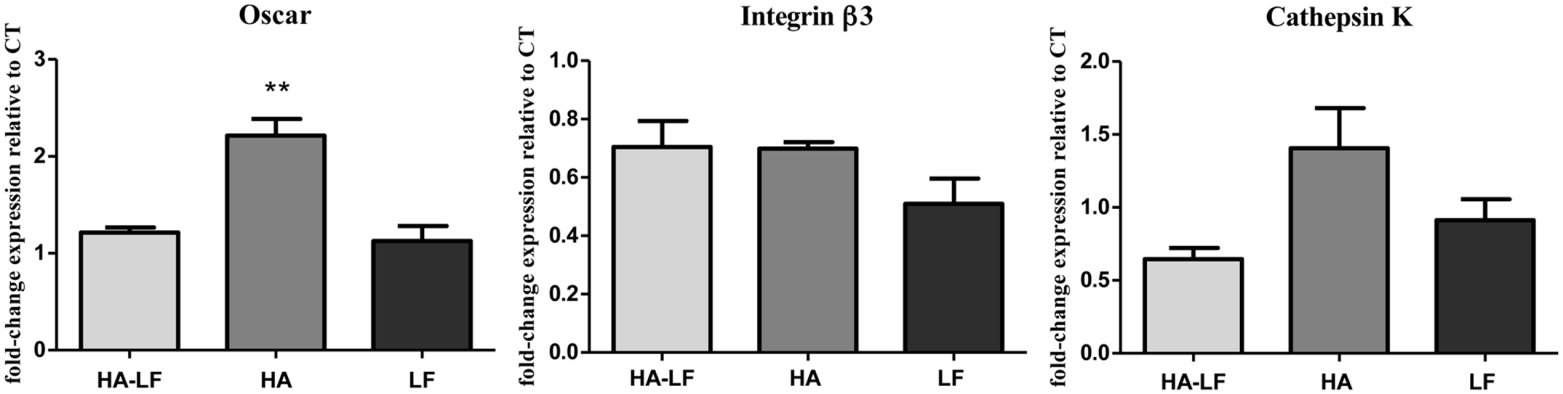
Co-cultured OCLs gene expression analysis. Relative quantification (2^-ΔΔCt^) of gene expression after 7 days of OCLs co-cultured with OBs grown in direct contact with all the tested samples. The graphs show the average and standard error of the technical triplicate of Oscar, Itgβ3 and CtsK, respect to the expression of the cells only, used as a control. Statistical significant difference among the samples is indicated in the graph: **p≤0.01.

The quantification of co-cultured OCLs related gene shown that HA-LF and LF induced a significant down-regulation of Oscar (p<0.01) and CtsK, even if no statistical difference was found, compared to HA; on the contrary, HA-LF seems not exert any effects on Itgβ3 relative expression (Fig 6).

## Discussion

In our previous work, it was demonstrated that the coupling of HA and LF leads to a combined effect in the induction of osteogenic differentiation of mesenchymal stem cells (MSCs) [20]. The present study should be considered a deeper biological characterization of HA functionalized with LF and a more detailed evaluation of their combined effects on the regulation of bone homeostasis. As well known, bone remodeling and regeneration are processes finely regulated and the intercellular communication within the bone microenvironment, carried out by all the bone cells, is a critical step for maintaining or restoring the normal bone physiology [30, 31]. For these reasons, potential direct and indirect effects of HA-LF system in the induction of pre-osteoblast cell line and in the inhibition of osteoclasts activity have been evaluated.

In our prior work, HA loaded with different amount of LF has been tested and it has been shown that the most promising sample was the HA loaded with 6.6 wt% of LF, supplied to the cells culture at 10 μg/ml concentration of attached LF (Table 1). The amount of 6.6 wt% of LF allowed to form a single layer of protein on HA surface with a better molecular orientation and higher bioavailability of LF to cells. Moreover interestingly, our previous results have pointed out that the 10 μg/ml of attached LF onto HA, that correspond to the physiological concentration of LF *in vivo*, showed better biological effects on MSCs than the 100 μg/ml of attached LF, concentration of LF typical of sepsis or inflammatory conditions *in vivo*, since the binding of LF to HA increased the bioavailability of this anabolic factor to the cells, reducing the concentration needed [20]. Therefore, in this work HA-LF was tested using the best conditions previously reported and the experiments were focused on the potential combined effect of this functionalized biomaterial on the others cellular components of the bone tissue (i.e. OBs and OCLs).

The results obtained by OBs grown in direct contact with HA-LF, demonstrated that the coupling of LF and HA leads to an inductive effect in term of viability and expression of OBs related genes. In terms of viability, it was found a decrease induced by HA sample only after 7 days of culture (Fig 1a), which disappeared after 14 days. In spite of the well-known HA biocompatibility, it is reasonable to hypothesize that such high concentration of material (141.5μg/ml) in a 2D *in vitro* model can lead to a weak reduction of cells viability [32]. Along with cell viability analysis, the HA-LF sample significantly decreased the apoptotic effect shown by HA only (Fig 1b), indicating that LF attached onto HA not only maintains its anabolic function, but also increase the bioactivity of HA nanocrystals.

Our results suggested that apoptosis is responsible for the decrease in OBs viability induced by biomaterial after 7 days of culture, probably due to the high nanocrystals concentration and content of intracellular calcium [32, 33]. Interestingly, also the induction of apoptosis of OBs by HA disappeared after 14 days of culture although the HA nanocrystals degradation at pH 7.4 (Hepes buffer 0.1 M solution), evaluated monitoring the Ca^2+^ ions released by Inductively coupled plasma optical emission spectrometry (ICP), showed a good HA stability in physiological conditions since only the 11±1% of total Ca^2+^ ions was released after 14 days (data not showed). These evidences indicates that probably longer time of culture allowed the OBs to restore the more physiological conditions even the HA stimuli persisted.

Noteworthy, HA-LF system showed an important effect in the induction of Osterix and IBSP, well known markers of osteoblast differentiation and activity [34–36]. The relative expression of these genes was double compared to the expression of the same genes in HA and LF samples, indicating that HA-LF has a synergistic effect on OBs expression of the genes involved in differentiation and production of bone matrix. As demonstrated in a previous study, LF has a strong affinity for HA surface and no LF has been released up to 14 day [20]. This finding indicated that the inductive effect observed on OBs was the results of the combined action of HA and LF.

Regarding the regulation of OCLs, it has been investigated the direct and indirect effect exert by HA-LF on OCLs differentiation and activity by culturing sRANKL-treated RAW 264.7 cells in direct contact with the samples (HA-LF, HA and LF) or in co-culture with OBs cultured with the samples.

The evaluation of large and multinucleated cells positive for tartrate-resistant acid phosphatase, detected by TRAP assay, indicated that cells were grown regularly during the experiments and confirming osteoclasts differentiation in our model. In Fig 3, one representative field of each sample has been shown and it was observed that the presence of LF, both adsorbed onto HA and free, seems to reduce the OCLs formation, confirming the literatures [37, 38].

In order to obtain quantitative results, the relative expression of OCLs related genes was detected by qPCR. The analyses showed that, in OCLs culture, only Itgβ was significantly down-regulated by HA-LF and LF compared to HA (Fig 4), while no differences in expression of Oscar and CtsK were found.

On the contrary, the results of co-cultured OCLs showed that the effects of LF were mediated by OBs and consist in the down-regulation of Oscar and Cathepsin K genes compared to HA, without affecting the Itgβ3 relative expression (Fig 6).

Interestingly, in both culture conditions, direct contact with biomaterials and OBs/OCLs co-culture, HA-LF showed the same down-regulatory effects on the genes tested exerted by free LF supplied to the cells, indicating that LF attached onto HA nanocrystal not only maintained its effect, but it can also inhibits the well-known stimulatory effect of HA on OCLs [39] directly by down-regulation of Itgβ3 and indirectly by down-regulation of Oscar and Cathepsin K.

It is reasonable to hypothesize that the increasing of the Itgβ3 relative expression induced by HA nanocrystals was related to the direct interaction between cells and biomaterial. *In vivo*, the interactions between osteoclasts and extracellular matrix (ECM) are thought to be mediated by integrin, especially integrin β3, which is generally considered as the major receptor in osteoclasts mediating signaling cascades involved in OCLs adhesion to the ECM, motility and activity [40–42]. Differently, Oscar and CtsK relative expression seems to be sensitive only to the OBs stimuli induce by HA-LF presence. Oscar is a OCLs associated receptor, its expression follows TRAP during osteoclasts differentiation [43]; CtsK is highly and quite selectively expressed in osteoclasts and it is a key protease in degradation of bone matrix molecules [44]. Therefore, the indirect effect of HA-LF in the expression of these genes confirmed that HA-LF acts as OCLs inhibitor. Moreover, this effect, mediated by OBs, seems to implicate the OPG/RANKL molecular pathway which is one of the principal signaling pathway involved in OBs/OCLs intracellular crosstalk [45]. As shown in Fig 4, the ratio of OPG/RANKL was higher in HA-LF and LF compared to HA, indicating that the presence of LF, both free and attached onto HA nanocrystals, stimulated the OBs to produce OPG. This soluble factor exerts its function by blocking the RANKL—RANK receptor interaction and it acts to antagonize the formation and survival of osteoclasts [46–48].

## Conclusions

In conclusion, these results confirm that HA and LF can act in synergism when coupled together as anabolic factor for osteoblasts differentiation and activity and, at the same time, they exert an important regulatory role in the inhibition of osteoclasts activity.

This *in vitro* study, aimed to lay the foundations for the future development of new generation of ideal HA-based bone graft substitutes integrating and carrying active biomolecules, such as LF, which made the biomaterials more bioactive.

The functionalization of HA nanocrystals with LF can (i) increase the local concentration of LF in the site of interest reducing the concentration needed compared to the oral administration; (ii) prolong its residence time and prevent early degradation and clearance and (iii) minimize patient discomfort and the serious side effects often associated to the oral administration of classic anti-osteoporotic drugs [49]. This approach could be used for several bone-related pathologies opening brilliant prospective for the future use in tissue engineering and regenerative medicine applications.
